# Achieving compliance with the International Health Regulations by overseas territories of the United Kingdom of Great Britain and Northern Ireland 

**DOI:** 10.2471/BLT.14.137828

**Published:** 2014-09-09

**Authors:** Esther L Hamblion, Mark Salter, Jane Jones, on behalf of the UK Overseas Territories and Crown Dependencies IHR Project Group

**Affiliations:** a Centre for Infectious Disease Surveillance and Control, Public Health England, 61 Colindale Avenue, London, NW9 5EQ, England.; b Global Health, Public Health England, London, England.

## Abstract

The 2005 International Health Regulations (IHR) came into force for all Member States of the World Health Organization (WHO) in June 2007 and the deadline for achieving compliance was June 2012. The purpose of the IHR is to prevent, protect against, control – and provide a public health response to – international spread of disease. The territory of the United Kingdom of Great Britain and Northern Ireland and that of several other Member States, such as China, Denmark, France, the Netherlands and the United States of America, include overseas territories, which cover a total population of approximately 15 million people. Member States have a responsibility to ensure that all parts of their territory comply with the IHR. Since WHO has not provided specific guidance on compliance in the special circumstances of the overseas territories of Member States, compliance by these territories is an issue for self-assessment by Member States themselves. To date, no reports have been published on the assessment of IHR compliance in countries with overseas territories. We describe a gap analysis done in the United Kingdom to assess IHR compliance of its overseas territories. The findings and conclusions are broadly applicable to other countries with overseas territories which may have yet to assess their compliance with the IHR. Such assessments are needed to ensure compliance across all parts of a Member States’ territory and to increase global health security.

## Résumé

Le Règlement sanitaire international de 2005 (RSI) est entré en vigueur pour tous les États membres de l'Organisation mondiale de la Santé en juin 2007, et la date limite pour sa mise en conformité était juin 2012. L'objectif du RSI est de prévenir, de protéger, de contrôler – et d'apporter une réponse de santé publique – à la propagation internationale des maladies. Le territoire du Royaume-Uni de Grande-Bretagne et d'Irlande du Nord et celui d'autres États membres, comme la Chine, le Danemark, la France, les Pays-Bas et les États-Unis d'Amérique, se composent de territoires d'outre-mer, lesquels couvrent une population totale d'environ 15 millions d'habitants. Les États membres ont la responsabilité de veiller à ce que toutes les parties de leur territoire se conforment au RSI. Étant donné que l'OMS ne fournit pas d'orientation spécifique concernant la conformité dans les circonstances spéciales des territoires d'outre-mer des États membres, leur conformité est une question d'auto-évaluation par les États membres eux-mêmes. À ce jour, aucun rapport n'a été publié sur l'évaluation de la conformité au RSI dans les pays possédant des territoires d'outre-mer. Nous décrivons une analyse des lacunes effectuée au Royaume-Uni pour évaluer la conformité au RSI de ses territoires d'outre-mer. Les résultats et les conclusions sont largement applicables aux autres pays possédant des territoires d'outre-mer, qui peuvent cependant évaluer leur propre conformité au RSI. Ces évaluations sont nécessaires pour veiller à la conformité dans toutes les parties du territoire d'un État membre et pour augmenter la sécurité sanitaire mondiale.

## Resumen

El Reglamento Sanitario Internacional 2005 (RSI) entró en vigor para todos los Estados miembros de la Organización Mundial de la Salud (OMS) en junio de 2007 con junio de 2012 como fecha límite para lograr el cumplimiento. El objetivo del RSI es prevenir, proteger, controlar y proporcionar una respuesta de salud pública a la propagación internacional de enfermedades. El territorio del Reino Unido de Gran Bretaña e Irlanda del Norte y otros Estados miembros como China, Dinamarca, Francia, los Países Bajos y los Estados Unidos de América cuentan con territorios de ultramar que abarcan una población total de aproximadamente 15 millones de personas. Los Estados miembros tienen la responsabilidad de garantizar que todos sus territorios cumplan con el RSI. Puesto que la OMS no ha proporcionado orientación específica sobre el cumplimiento para las circunstancias especiales de los territorios de ultramar de los Estados miembros, el cumplimiento por parte de estos territorios es un problema que los propios Estados miembros tienen que evaluar. Hasta la fecha no se han publicado informes sobre la evaluación del cumplimiento del RSI en los países con territorios de ultramar. Describimos un análisis de las deficiencias realizado en el Reino Unido con objeto de evaluar el cumplimiento del RSI de sus territorios de ultramar. Los resultados y conclusiones son ampliamente aplicables a otros países con territorios de ultramar que quizá aún tengan que evaluar su cumplimiento con el RSI. Dichas evaluaciones son necesarias para asegurar el cumplimiento en todos los territorios de los Estados miembros y para aumentar la seguridad sanitaria mundial.

## ملخص

دخلت اللوائح الصحية الدولية لعام 2005 حيز التنفيذ لدى جميع الدول الأعضاء في منظمة الصحة العالمية في حزيران/ يونيو 2007، وكان حزيران/ يونيو 2012 آخر موعد محدد لتحقيق الامتثال. وتهدف اللوائح الصحية الدولية إلى توقي انتشار الأمراض على الصعيد الدولي والحماية منه ومكافحته وتوفير استجابة صحة عمومية لهذا الانتشار. ويتضمن إقليم المملكة المتحدة لبريطانيا العظمى وأيرلندا الشمالية وإقليم عدة دول أخرى من الدول الأعضاء مثل الصين والدانمرك وفرنسا وهولندا والولايات المتحدة الأمريكية أقاليم خارجية يبلغ إجمالي سكانها 15 مليون شخص تقريباً. وتتحمل الدول الأعضاء مسؤولية التأكد من امتثال جميع أجزاء الإقليم التابع لها للوائح الصحية الدولية. ونظراً لأن منظمة الصحة العالمية لم تقدم إرشادات معينة عن الامتثال في الظروف الخاصة للأقاليم الخارجية للدول الأعضاء، فإن امتثال هذه الأقاليم مسألة تقييم ذاتي من قبل الدول الأعضاء أنفسها. ولم يتم حتى الآن نشر أي تقارير حول تقييم الامتثال للوائح الصحية الدولية في البلدان التي تتبعها أقاليم خارجية. ونقوم بوصف تحليل الثغرات الذي أجري في المملكة المتحدة لتقييم امتثال الأقاليم الخارجية التابعة لها للوائح الصحية الدولية. ويمكن على نحو واسع تطبيق النتائج والاستنتاجات على البلدان الأخرى التي تتبعها أقاليم خارجية ما زال يتعين تقييم امتثالها للوائح الصحية الدولية. وتوجد حاجة لهذه التقييمات لضمان الامتثال في جميع أجزاء أي إقليم يتبع الدول الأعضاء ولزيادة الأمن الصحي العالمي.

## 摘要

2007年6月,2005年《国际卫生条例》(IHR)在世界卫生组织(WHO)所有会员国生效,而合规实现的最后期限是2012年6月。IHR旨在预防、防范、控制疾病的国际传播并提供公共卫生应对措施。英国与中国、丹麦、法国、荷兰和美国等其他几个成员国的领土包括海外领土,人口总数约为1500万人。成员国有责任确保所有领土遵守IHR。因为WHO还没有提供成员国海外领土这种特殊情况下合规的具体指导,这些领土的合规是成员国自我评价的问题。到目前为止,有海外领土的国家还没有发表评估IHR合规的报告。我们描述在英国完成的一项评估其海外领土IHR合规性的差距分析。这些结果和结论广泛适用于其他有海外领土且可能尚未评估《国际卫生条例》合规性的国家。确保成员国所有领土合规性并提高全球卫生安全需要这种评估。

## Резюме

Международные медико-санитарные правила (ММСП), принятые в 2005 г., вступили в силу для всех государств-членов Всемирной организации здравоохранения (ВОЗ) в июне 2007 года с планируемым сроком достижения соответствия в июне 2012 года. Целью ММСП является предотвращение, защита и борьба, включая меры общественного здравоохранения, с международным распространением болезней. Территории Соединенного Королевства Великобритании и Северной Ирландии, а также некоторых других государств-членов ВОЗ, таких как Китай, Дания, Франция, Нидерланды и США, включают заморские территории, где проживает около 15 млн. человек. Государства-члены несут ответственность за соблюдение ММСП во всех частях своей территории. Поскольку ВОЗ не предоставила конкретных указаний о соблюдении данных правил в особых условиях на заморских территориях своих государств-членов, соблюдение правил ММСП на этих территориях является делом самостоятельной оценки для всех государств-членов ВОЗ. На сегодняшний день не было опубликовано никаких сообщений по оценке соответствия ММСП в странах с заморскими территориями. В данной статье описывается анализ пробелов, проведенный в Великобритании, с целью оценки соблюдения ММСП на заморских территориях этой страны. Полученные результаты и выводы могут широко применяться для других стран с заморскими территориями, которым, возможно, еще предстоит оценить свое соответствие ММСП. Такие оценки необходимы для обеспечения соблюдения медико-санитарных правил во всех частях территорий государств-членов ВОЗ и

## Introduction

The 2005 International Health Regulations [IHR (2005)] came into force in June 2007 for 194 countries, including all Member States of the World Health Organization (WHO).^1^ The deadline for compliance was June 2012. Nine Member States, including China, Denmark, France, the Netherlands, the United Kingdom of Great Britain and Northern Ireland and the United States of America, have overseas territories with a total population of more than 15 million people (Table 1).^2^^,^^3^ These territories fall under the jurisdiction and sovereignty of the mainland yet are often self-governing and have their own legal and public health systems. However, each Member State has a responsibility to ensure that all parts of its territory comply with the IHR (2005).

**Table 1:** Population of overseas territories belonging to Member States of the World Health Organization, 2009–2014

Member State	Estimated population of overseas territories^2^^,^^3^
Australia	4 340
China	7 660 400
Denmark	105 000
France	2 583 417
Netherlands	518 966
New Zealand	12 661
Norway	1 890
United Kingdom	248 771
United States	4 100 650
**Total population**	**15 236 095**

The epidemiology of disease in overseas territories has been reported to differ from that in their respective mainland territories.^4^^,^^5^ For example, epidemics of tropical diseases that are largely absent from mainland Europe persist in the Caribbean overseas territories of some European nations.^5^ Some overseas territories receive a large number of holidaymakers from their respective mainlands, which increases the risk of disease being imported in both directions. It is therefore of paramount importance that all Member States with overseas territories are aware of the situation regarding full compliance with the IHR (2005).

The purpose of the IHR (2005) is “to prevent, protect against, control – and provide a public health response to – the international spread of disease in ways that are commensurate with and restricted to public health risks and which avoid unnecessary interference with international traffic and trade.”^1^ Under the revised regulations, Member States have much broader obligations to build national capacity for surveillance and response in the event of a public health emergency of international concern and to share information about such emergencies. The regulations include a code of conduct for notification and response.

The IHR (2005) requires countries to assess the ability of existing national structures, capacities and resources to meet minimum requirements for public health surveillance and response. These assessments, together with the consequent development of plans to ensure compliance with the IHR (2005), were supposed to be completed by June 2012. However, fewer than 20% of Member States met this deadline.^6^

No specific guidance on compliance with the IHR (2005) has been provided by WHO for the special circumstances of Member States with overseas territories, which indicates that compliance by overseas territories should be assessed by Member States themselves. To our knowledge, no reports have been published to date on the assessment of IHR compliance in countries with overseas territories.

The aims of this paper were: (i) to provide an overview of a gap analysis done to assess IHR compliance of the United Kingdom’s overseas territories and to identify appropriate measures that could be taken to ensure compliance by June 2014; and (ii) to discuss the policy implications of the findings of the gap analysis, which may be relevant to other countries with overseas territories.

## Compliance by overseas territories

Although mainland United Kingdom was almost fully compliant with the IHR by June 2012, it was recognized that compliance by the United Kingdom’s overseas territories and crown dependencies required further assessment. An extension of the deadline for IHR compliance to June 2014 was therefore obtained.

**Fig. 1:**
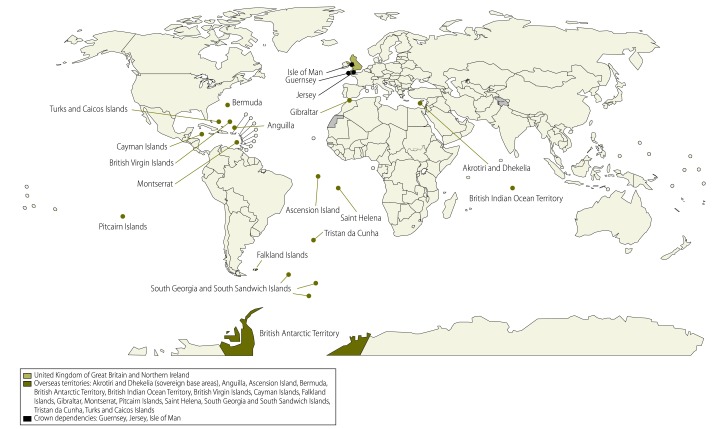
The United Kingdom of Great Britain and Northern Ireland and its overseas territories and crown dependencies, 2014

The United Kingdom has 16 overseas territories and three crown dependencies (Fig. 1). These territories and dependencies are heterogeneous in terms of: (i) geography and environmental conditions (one crown dependency is located less than 70 miles from mainland United Kingdom, whereas others are some of the most remote islands in the world); (ii) population size, which ranges from zero in uninhabited territories to 95 000; and (iii) income (the per capita gross domestic product ranges from less than one tenth to nearly double that of the United Kingdom).^3^ These factors all influence the public health situation in the territories and dependencies.^4^^,^^7^ Between the introduction of the IHR (2005) in June 2007 and the end of December 2010, only one public health emergency of international concern was declared by WHO: pandemic influenza A (H1N1) in 2009. However, other events occurred in the United Kingdom’s overseas territories and crown dependencies that fell within the remit of the IHR (2005), including a measles outbreak in Saint Helena in 2010 and an outbreak of respiratory illness in Tristan da Cunha in 2007.^8^

## Assessing compliance

A structured, self-assessment questionnaire on compliance with the IHR (2005) was designed for the United Kingdom’s overseas territories and crown dependencies following a literature search on the implementation of, and assessment of compliance with, the regulations in other countries. The format of the questionnaire was based on a self-assessment tool proposed by WHO.^9^^–^^11^ However, since the level of detail in this tool exceeded the requirements laid out in the IHR, the questionnaire was used for collecting information but did not serve as the standard for assessing compliance.

For 15 of the United Kingdom’s 19 overseas territories and crown dependencies, structured telephone interviews were carried out with individuals named on the United Kingdom’s national focal point distribution list. These individuals were often directors of public health or chief medical officers but colleagues they deemed necessary to answer the questions posed, such as epidemiologists, emergency planning officers and food safety experts, were also interviewed. Interviewees were encouraged to complete the self-assessment questionnaire before the interview. In the four remaining territories, no telephone interviews were carried out but further discussions were conducted by email, when necessary, after the self-assessment questionnaires had been completed. 

In our assessment, compliance with IHR (2005) core capacity areas was assessed using descriptions of these areas in the IHR (2005) themselves rather than the higher level of detail included in the self-assessment tool provided by WHO. This approach is consistent with that adopted for mainland United Kingdom. Compliance was assessed using a traffic light system: (i) red for areas in which the overseas territory or crown dependency was not compliant; (ii) amber for areas in which there was broad compliance but which would benefit from further work to strengthen some components of the core capacity area; and (iii) green for areas in which there was full compliance.

**Fig. 2:**
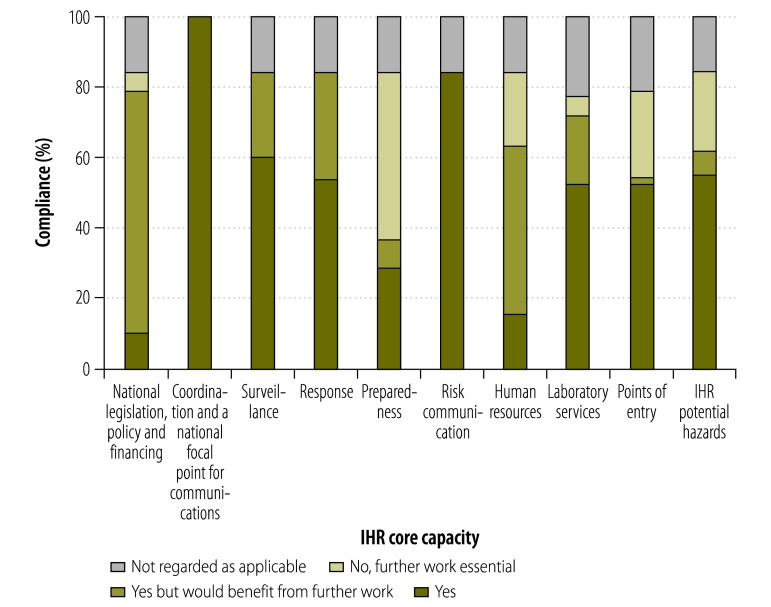
Proportion of overseas territories and crown dependencies that comply with International Health Regulations (2005) core capacities, United Kingdom of Great Britain and Northern Ireland, 2013

We found that all territories and dependencies were largely compliant in the IHR (2005) core capacity areas (Fig. 2). For three territories – British Antarctic Territory, British Indian Ocean Territory and South Georgia and the South Sandwich Islands – questionnaire responses indicated that the majority of the core capacity areas did not apply because the territories do not have indigenous populations. However, each had a national focal point for communications and a contact for coordinating activities associated with the IHR (2005). Five other overseas territories and crown dependencies were broadly compliant in all core capacity areas, where applicable, but would benefit from development work to strengthen public health systems.

IHR: International Health Regulations (2005).

## Implications for countries

Our assessment of the compliance of the United Kingdom’s overseas territories and crown dependencies with the IHR (2005) identified common areas where further work had to be undertaken to ensure compliance and where the ability of these territories and dependencies to detect and respond to a public health emergency could be improved. These findings will be applicable to other countries with overseas territories.

First, the majority of the United Kingdom’s overseas territories and crown dependencies needed to update emergency response plans to comply with Annex 1A [item 6(g)] of the IHR (2005).^1^ This is also likely to be the case for other overseas territories. Any update to existing plans must: (i) incorporate an all-hazards approach (i.e. be able to deal with zoonosis, food safety and chemical and radionuclear events); (ii) identify supply lines for stockpiles of priority drugs, protective equipment for personnel, chemical antidotes to toxins and emergency supplies to deal with a radiation hazard; and (iii) identify resources outside the territory that can boost surge capacity (i.e. the ability to deal with a surge in cases) in the event of a public health emergency – this may involve the creation of appropriate memoranda of understanding with other parties to define both the relationship and expectations.

Although all of the United Kingdom’s overseas territories and crown dependencies had the technical ability to respond to a public health emergency or public health emergency of international concern, the majority did not have the necessary expertise in zoonosis, food safety or dealing with chemical or radionuclear events because of their population size and geographical isolation. This is likely to be the case for all overseas territories, particularly as it has been observed that, in much of the world, the surveillance capacity for public health incidents involving chemical or radionuclear material is underdeveloped or nonexistent.^12^ To achieve compliance, all overseas territories should compile a directory of expertise, which includes memoranda of understanding with appropriate external organizations, to bridge the gap in expertise and surge capacity – collaboration is a key aspect of the IHR (2005).^13^ In addition, an inventory of potential hazards should be compiled and training on all hazards should be given.

The ability to assess points of entry is regarded as critical by WHO and it has been noted that meeting IHR (2005) obligations with regard to points of entry is a universal challenge.^14^ For many overseas territories it may not be appropriate to designate a point of entry for IHR purposes as many ports and airports in these territories are unlikely to have the facilities or resources to deal with a public health emergency of international concern. The United Kingdom’s overseas territories were therefore recommended not to designate points of entry for IHR purposes. A number of UK overseas territory ports do have the capacity to issue ship sanitation certificates and a list of these ports has been provided to WHO in accordance with article 20 (item 3) of the IHR (2005).^1^ Furthermore it may be technically challenging to ensure that all core capacity requirements have been met (Box 1).

Box 1 **Core capacity requirements for designated points of entry, Annex 1B of the International Health Regulations (2005)^1^**

**1. At all times. The capacities:**

a) to provide access to:

- appropriate medical services, including diagnostic facilities to allow prompt assessment and care of ill travellers, and
- adequate staff, equipment and premises;

b) to provide access to equipment and personnel for the transport of ill travellers to an appropriate medical facility;

c) to provide trained personnel for the inspection of conveyances;

d) to ensure a safe environment for travellers using points-of-entry facilities, including:

- potable water supplies
- eating establishments
- flight catering facilities
- public washrooms and
- appropriate solid and liquid waste disposal services and other potential risk areas, by conducting inspection programmes as appropriate; and

e) to provide, as far as practical, a programme and trained personnel for the control of vectors and reservoirs in and near points of entry.

**2. For responding to a public health emergency of international concern. The capacities:**

a) to provide appropriate public health emergency response by establishing and maintaining a public health emergency contingency plan, including

- the nomination of a coordinator, and
- contact points for the relevant point of entry, public health and other agencies and services;

b) to provide assessments of and care for affected travellers or animals by:

- establishing arrangements with local medical and veterinary facilities for their isolation, treatment and other support services that may be required;

c) to provide appropriate space, separate from other travellers, to interview suspect or affected persons;

d) to provide for the assessment and, if required, quarantine of suspect travellers, preferably in facilities away from the point of entry;

e) to apply recommended measures to:

- disinsect
- derat
- disinfect
- decontaminate, or
- otherwise treat baggage, cargo, containers, conveyances, goods or postal parcels including, when appropriate, at locations specially designated and equipped for this purpose;

f) to apply entry or exit controls for arriving and departing travellers; and

g) to provide access to:

- specially designated equipment, and
- to trained personnel with appropriate personnel protection for the transfer of travellers who may carry infection or contamination.

The IHR (2005) recognize that Member States have the sovereign right to legislate and implement legislation. Nevertheless, article 3 states that legislation should uphold the purpose of the regulations.^1^ The majority of the United Kingdom’s overseas territories and crown dependencies were technically compliant with national legislation and requirements. However, the legislation was often outdated, which could also be the case for other overseas territories. Consequently, legislation should be reviewed to facilitate full and efficient implementation of the regulations, for example, to include an all-hazards approach. The lack of skilled personnel for reviewing and drafting legislation and the lack of finance are recognized as barriers to this task, therefore advice and support from appropriate experts may be required. The allocation of funding to support the implementation of the IHR (2005) is not explicitly a requirement for compliance, but if there is no specific budget – which was the case for most of the United Kingdom’s overseas territories and crown dependencies – it is difficult to complete the tasks required to achieve compliance.

Effective surveillance is paramount to an efficient and successful public health system and an effective capability for surveillance and response minimizes the risk of disease spreading across borders.^6^ Although all the United Kingdom’s overseas territories and crown dependencies were able to carry out routine surveillance as mandated under article 5 of the IHR (2005)^1^ (which concerns the detection, assessment, notification and reporting of events), some need to enhance their capabilities. The methods used to detect and report events will vary according to the nature of the overseas territory and its circumstances.

The ability to respond to a public health emergency is not strictly a requirement of the IHR (2005). Nevertheless, in order to respond effectively to both public health emergencies of international concern and other public emergencies, overseas territories need to develop plans to ensure a rapid response to all hazards, enlisting external support as appropriate. Moreover, WHO recommends that standard operating procedures be developed for case management in priority infectious diseases and for chemical and radionuclear events. Such procedures were generally available for infectious diseases, but not for chemical or radionuclear events in most of the United Kingdom’s overseas territories and crown dependencies. The use of standard case definitions increases the specificity of reporting and improves the comparability of events in different geographical areas.^15^ The majority of overseas territories would benefit from developing guidelines for the management of poisoning and chemical and radionuclear events. Observations in the United Kingdom’s overseas territories and crown dependencies indicate that it would probably be difficult to sustain a public health response during a disease outbreak. 

It has been recognized that one of the main challenges in implementing the IHR (2005) is the presence of gaps in human resources^14^ and this was reflected in our assessment. Although there is no requirement for personnel to have a specific level of skill, WHO has recognized that a skilled public health workforce is essential for an effective health system.^16^^,^^17^ Self-assessments in these territories and dependencies indicated that there was a relative lack of staff with expertise in public health surveillance and response because of the small populations and remote locations. This is likely to be true for other overseas territories. It is also important to ensure that the requirements of the IHR (2005) can be satisfied not only during public health emergencies but also, for example, when personnel are on leave. In addition, where the turnover of staff is high, it is important to ensure that individuals with the necessary skills continue to be available. Training, perhaps remotely through web-based systems, is needed so that staff know how to detect, and respond to, public health emergencies.

Although none of the United Kingdom’s overseas territories or crown dependencies were able to test for all priority infectious hazards, gaps in testing capacity were addressed by collaboration with laboratories with additional resources outside the territory – this solution has previously been identified as a way of complying with the IHR (2005).^15^ Similar gaps are probably present in all overseas territories. Moreover, because of their remoteness, these territories will experience delays in processing samples when they are sent outside the territory. Consequently, it is recommended that overseas territories develop service agreements with collaborating laboratories so that the expected timelines are clear. Some of the United Kingdom’s overseas territories and crown dependencies encountered carriers that were concerned about transporting potentially hazardous samples by air. The safe and effective transportation of samples is a recognized problem in many parts of the world.^14^ It is recommended, therefore, that service agreements addressing the requirements of the International Air Transport Association for the safe shipment of biological specimens be drawn up and that triple packaging materials be readily available within overseas territories and crown dependencies.

Some questionnaire respondents felt that laboratory biosafety and security were not adequate. Further work should be undertaken on this issue even though it is not strictly necessary for compliance with the IHR (2005).

## Conclusion

Smaller countries and territories face challenges in complying with the IHR (2005). However, the process provides them with an opportunity to build, strengthen and maintain core capacities for public health surveillance and response.^18^ In addition, any assessments carried out can be used to provide baseline data for monitoring future improvements in public health systems. In the United Kingdom’s overseas territories, there was a good understanding of what is required to implement the IHR (2005) and many territories had already undertaken a substantial amount of work to ensure compliance. Nevertheless, knowledge of the regulations and their implications was not strong in some overseas territories, particularly in those where personnel changed each year. This is likely to be the case in many overseas territories.

Successful implementation of the IHR (2005) necessarily involves a realistic assessment of shortcomings.^14^ Globally, it is assumed that all parts of the territory of WHO Member States are compliant with the regulations with regard to public health surveillance and response. However, this assumption is not realistic for overseas territories because they are dispersed geographically over a vast area. Although the assessment we undertook for the United Kingdom’s overseas territories fulfilled the purpose of evaluating compliance with IHR (2005),^16^ it relied on self-assessment rather than on independent evaluation, which may have resulted in bias. To ensure that all parts of the territory of WHO Member States comply with IHR (2005), countries with overseas territories should carry out similar assessments. Subsequently, all overseas territories should develop action plans to address areas in which they are not currently compliant. There is, however, no single model appropriate for all overseas territories because they vary greatly in geography, population, health service capabilities and disease epidemiology.

In conclusion, although overseas territories are likely to be largely compliant with IHR (2005), as the United Kingdom’s overseas territories were, there will be gaps that need to be bridged to ensure global public health security.

## References

[1] Resolution WHA58.3: revision of the International Health Regulations. In: Fifty-eighth World Health Assembly, Geneva, 16–25 May 2005. WHA58/2005/REC/1. Geneva: World Health Organization; 2005. Available from: http://apps.who.int/gb/ebwha/pdf_files/WHA58-REC1/english/A58_2005_REC1-en.pdf [cited 2014 Aug 26].

[2] Overseas territories, dependent areas, and disputed territories [Internet]. Bangkok: One World – Nations Online; 2014. Available from: http://www.nationsonline.org/oneworld/territories.htm [cited 2014 Jul 29].

[3] The world factbook [Internet]. Washington: Central Intelligence Agency; 2014. Available from: https://www.cia.gov/library/publications/the-world-factbook/index.html[cited 2014 Jul 29].

[4] Jones J, Gastellu-Etchegorry M, Stenz FK, Baudon C, Bloem SJ, Bondonneau M, et al. Epidemiology, surveillance and control of infectious diseases in the European overseas countries and territories, 2011. Euro Surveill. 2011;16(29) pii: 19923. 21801693

[5] Gossner CM, Van Cangh T, Coulombier D. Public health in the European overseas countries and territories: new perspectives for Europe. Euro Surveill. 2011;16(29) pii: 19920. 21801695

[6] Braden CR, Dowell SF, Jernigan DB, Hughes JM. Progress in global surveillance and response capacity 10 years after severe acute respiratory syndrome. Emerg Infect Dis. 2013;19(6):864–9. 10.3201/eid1906.130192 23731871 PMC3713843

[7] Assessment of the needs of European overseas territories. Stockholm: European Centre for Disease Prevention and Control; 2009.

[8] International health Regulations. Activities of the UK national focal point from 15 June 2007 to December 2010. London: Health Protection Agency; 2011. Available from: http://webarchive.nationalarchives.gov.uk/20140714084352/http://www.hpa.org.uk/webc/HPAwebFile/HPAweb_C/1317132024882 [cited 2014 Sep 9].

[9] IHR monitoring framework: checklist and indicators for monitoring progress in the implementation of IHR core capacities in States Parties. Processes and outputs. Geneva: World Health Organization; 2007. Available from: http://www.who.int/ihr/Processes_of_IHR_Monitoring_framework_and_Indicators.pdf [cited 2014 Aug 26].

[10] Protocol for assessing national surveillance and response capacities for the International Health Regulations (2005). A guide for assessment teams. Geneva: World Health Organization; 2010. Available from: http://www.who.int/ihr/publications/who_hse_ihr_201007_en.pdf [cited 2014 Aug 26].

[11] IHR core capacity monitoring framework: checklist and indicators for monitoring progress in the implementation of IHR core capacities in States Parties. Geneva: World Health Organization; 2013. Available from: http://apps.who.int/iris/bitstream/10665/84933/1/WHO_HSE_GCR_2013.2_eng.pdf [cited 2014 Jul 29].

[12] Anema A, Druyts E, Hollmeyer HG, Hardiman MC, Wilson K. Descriptive review and evaluation of the functioning of the International Health Regulations (IHR) Annex 2. Global Health. 2012;8(1):1. 10.1186/1744-8603-8-1 22233652 PMC3313850

[13] Rodier G, Hardiman M, Plotkin B, Ganter B. Implementing the International Health Regulations (2005) in Europe. Euro Surveill. 2006;11(12):208–11. 17370971

[14] Katz RL, Fernandez JA, McNabb SJ. Disease surveillance, capacity building and implementation of the International Health Regulations (IHR[2005]). BMC Public Health. 2010;10 Suppl 1:S1. 10.1186/1471-2458-10-S1-S1 21143819 PMC3005569

[15] Nsubuga P, Nwanyanwu O, Nkengasong JN, Mukanga D, Trostle M. Strengthening public health surveillance and response using the health systems strengthening agenda in developing countries. BMC Public Health. 2010;10 Suppl 1:S5. 10.1186/1471-2458-10-S1-S5 21143827 PMC3005577

[16] Omaswa F. Human resources for global health: time for action is now. Lancet. 2008;371(9613):625–6. 10.1016/S0140-6736(08)60277-9 18295005

[17] Taboy CH, Chapman W, Albetkova A, Kennedy S, Rayfield MA. Integrated disease investigations and surveillance planning: a systems approach to strengthening national surveillance and detection of events of public health importance in support of the International Health Regulations. BMC Public Health. 2010;10 Suppl 1:S6. 10.1186/1471-2458-10-S1-S6 21143828 PMC3005578

[18] Oshitani H, Ailan L, Roces MC, Sian DT, Ken C, Kiedrzynski T. Implementing the new International Health Regulations in the Pacific – challenges and opportunities. Pac Health Dialog. 2005;12(2):135–43. 18181505

